# On the Accuracy of Fault Diagnosis for Rolling Element Bearings Using Improved DFA and Multi-Sensor Data Fusion Method

**DOI:** 10.3390/s20226465

**Published:** 2020-11-12

**Authors:** Qiang Song, Sifang Zhao, Mingsheng Wang

**Affiliations:** National Engineering Laboratory for Electric Vehicles, Beijing Institute of Technology (BIT), Beijing 100081, China; 3120185233@bit.edu.cn (S.Z.); 3120170198@bit.edu.cn (M.W.)

**Keywords:** bearing fault, detrended fluctuation analysis, fault diagnostics, linear discriminant analysis, multi-sensor data fusion

## Abstract

Rolling element bearings are widely employed in almost every rotating machine. The health status of bearings plays an important role in the reliability of rotating machines. This paper deals with the principle and application of an effective multi-sensor data fusion fault diagnosis approach for rolling element bearings. In particular, two single-axis accelerometers are employed to improve classification accuracy. By applying the improved detrended fluctuation analysis (IDFA), the corresponding fluctuations detrended by the local fit of vibration signals are evaluated. Then the polynomial fitting coefficients of the fluctuation function are selected as the fault features. A multi-sensor data fusion classification method based on linear discriminant analysis (LDA) is presented in the feature classification process. The faults that occurred in the inner race, cage, and outer race are considered in the paper. The experimental results show that the classification accuracy of the proposed diagnosis method can reach 100%.

## 1. Introduction

Nowadays, rotating machines play a major role in agricultural and industrial applications. These applications include wind generation, washing machines, electric vehicles, etc. Rolling element bearings are the key component of rotating machinery. The sudden failures of bearings would cause system outage. Monitoring the health status of bearings by collecting sensor signals can help diagnose already-developed faults, and the probability of further damage can be reduced [1]. The classification accuracy has increasingly become a concern in the fault diagnosis system of ball bearings. Therefore, many scholars have studied the feature extraction and the classifier design of bearing failures [2,3].

Currently, two types of sensor techniques are widely used in fault feature extraction. One is the current-based technique. Extensive studies on current signature analysis have been conducted for the bearing fault diagnosis of electric motors [4,5,6]. The extra torque ripple would be generated under bearing failure conditions, so the feature components of the current harmonics could be produced by the torque change [7]. The current-based technique is not applicable for all kinds of rotating machinery (such as aero-engine, gearbox, etc.), and the feature current harmonics is sensitive to the load fluctuation. The vibration-based technique is another commonly used approach for bearing fault feature extraction [7,8,9,10]. The rotating machinery is usually operated in the non-linear condition. Therefore, one difficulty in applying the vibration signature analysis is the nonstationary properties of the acquired signals. In recent years, several vibration signal analysis techniques have been developed for fault feature extraction, using frequency techniques [11,12], and time-frequency methods [13,14] to extract fault features. Fourier transform (FT), as the traditional frequency analysis tool, is widely used for feature extraction [15]. However, this approach is ineffective to analyze vibration signals. To overcome the hurdle, time-frequency methods have been developed. In general, the common time-frequency analysis tools include [16,17,18,19,20]: short-time Fourier transform (STFT), wavelet analysis (WA), empirical mode decomposition (EMD), Wigner-Ville distribution (WVD), and Hilbert-Huang transform (HHT). Compared with FT, STFT can be used to localize the transients, while the drawback of STFT is that the accuracy of extracting frequency information is limited [8]. WA is the most researched approach for bearing fault feature extraction, and is effective for bearing fault diagnostics. With the use of this method, the high resolution in time and frequency domains can be provided. However, the limitation of WA is that a basic wavelet function should be determined before analyzing the vibration signals. EMD is an effective approach for processing nonlinear and nonstationary signals. When EMD is applied for feature extraction, a vibration signal can be decomposed into many intrinsic mode functions (IMFs), the information of the analyzed signal would be contained in each IMF. The main drawback of EMD is the poor translation-invariant property. WVD is suitable for analyzing the single-component signal, however, because the window function is not involved, the result of WVD would be interfered by cross terms when analyzing multi-component signals. HHT is appropriate for transient signal detection, and successful applications of this signal processing method for fault feature extraction have been reported in [21,22,23]. Both frequency and time-frequency techniques rely on the identification of the frequencies present, which are then compared with models to predict which frequencies should be important in the presence of various faults [24].

As a calculation method of time series long-range correlation scale index, detrended fluctuation analysis (DFA) was first proposed to differentiate between local patchiness and long-range correlations in DNA sequences [25]. This tool can eliminate the external trend of signals effectively, and has been gradually applied in medicine, finance, meteorology, hydrology, and other fields. Several scholars have employed DFA to the processing of mechanical vibration signals. Moura et al. [24] use DFA for gear fault identification, and the fault vibration signals under different working conditions are distinguished effectively. Jiang et al. [26] employ DFA in feature extraction for gearbox fault diagnosis, and several combinations of the features are used for the classification of fault types. Wang et al. [27] present the analysis of the vibration time series of a gear system acquired by a piezoelectric acceleration transducer using DFA. In the literature, there are few studies on DFA applied for feature extraction in bearing fault diagnostics. Commonly, the least-squares method is employed for DFA to fit the fluctuation function and extract the fault features. However, the fitting effect of the least-squares method would become poor when the function is complex. Moreover, for one vibration sequence, DFA can only extract two fault features. The insufficient number of fault features would result in a reduction in the classification accuracy in the diagnosis application of multiple fault types. Therefore, improved detrended fluctuation analysis (IDFA) is proposed in this paper to make up for the deficiency of the DFA method for fault feature extraction.

The variation of the detrended fluctuations is a signature of the bearing fault type, and this signature can be classified by the classifier. In general, the classifiers for bearing fault diagnostics mainly include [28,29]: artificial neural networks (ANNs), support vector machines (SVMs), K-means clusters, fuzzy classifiers, and Bayesian algorithms. These classifiers have been used successfully in signal fault detection. Linear discriminant analysis (LDA) is a classical machine learning method, which was first proposed by Fisher in 1936, and is also known as Fisher linear discriminant analysis. The principle of LDA is simple: given a set of training samples, it tries to project the samples to a straight line, so that the projection points of the same class are as close as possible. When classifying new samples, it projects the data to the trained straight line, and then the category of the tested samples can be determined according to the location of the projection points. LDA has been widely used in face recognition [30], biomedical research [31], and induction motor fault diagnosis [32]. In this paper, LDA is chosen as a classification method to process the feature vectors.

In reviewing multi-sensor data fusion approaches reported in the literature, these methods can be classified into two types based on the sensor [33,34,35]. One is based on the information collected from various types of sensors, such as current, voltage, vibration, sound, and temperature, to data fusion and detect faults. In [34], a hybrid approach for fault signal classification is presented based on sensor data fusion by using the SVM and STFT techniques. This method can use the fault information collected by different kinds of sensors and have high classification accuracy. However, the complexity of data processing would be increased by collecting data from different types of sensors. A different number of the same kind of sensor is employed by another multi-sensor data fusion method. In [35], two fault features are developed to characterize the gear health conditions, and an adaptive neuro-fuzzy inference system is utilized to fuse all features collected from vibration sensors mounted on different locations. However, the accuracy of the diagnosis method is limited. In this work, the information obtained from vibration sensors installed in different positions is used for the data fusion. The proposed diagnosis method is developed by integrating the IDFA-based feature extraction and the multi-sensor data fusion-based LDA classifier design. Three faults of bearings are tested in this work: inner race fault, cage fault, and outer race fault. Experiments with different fault degrees are conducted to validate the effectiveness of the proposed method.

The main contributions of this paper include: (1) applying DFA for feature extraction of bearing faults, and the deficiencies of the DFA-based extraction approach are analyzed for the application of multiple fault type diagnosis. (2) The IDFA feature extraction method based on the polynomial fitting and particle swarm optimization (PSO) algorithm is presented to improve the defects of DFA in the application of bearing fault diagnosis. (3) Using the vibration data of two sensors to improve the accuracy of fault diagnosis, and a multi-sensor data fusion fault diagnosis method based on LDA is proposed.

The remainder of the paper is organized as follows. In Section 2, the proposed bearing fault diagnosis approach using IDFA and a multi-sensor data fusion method is described. Section 3 illustrates the IDFA-based feature extraction method. In Section 4, the multi-sensor data fusion approach and the methodology of the LDA algorithm are explained. The experimental setup and the diagnostic results are presented in Section 5. Finally, Section 6 concludes the paper.

## 2. Fault Diagnosis Based on IDFA and Multi-Sensor Fusion

**Figure 1:**
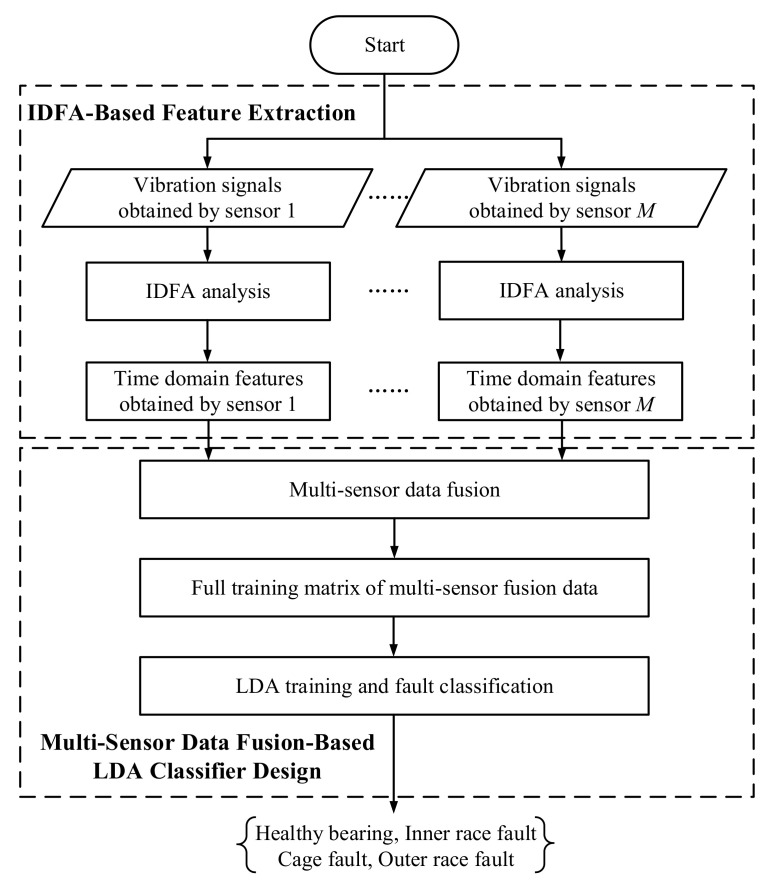
Scheme of the proposed fault diagnosis method for rolling element bearings.

The scheme of the proposed multi-sensor data fusion-based fault diagnosis approach is presented in Figure 1. As shown in Figure 1, two steps are included: IDFA-based feature extraction and multi-sensor data fusion-based LDA classifier design. In the first step, acceleration sensors mounted on different locations are employed for the acquisition of vibration signals. The detrended fluctuation function of each signal is then calculated by IDFA, and the time-domain features obtained from vibration sensors are extracted. In the second step, these time-domain features are reconstructed into one feature vector to obtain the full training matrix of the LDA classifier. Finally, the type of bearing fault can be identified by using the trained LDA classifier.

## 3. IDFA-Based Feature Extraction

### 3.1. Methodology of DFA

On assumption that any noise present in the signal is non-correlated, time-series analysis methods can be applied to identify properties of variation signals. In order to analyze the correlated components in a time-series signal, fractional Brownian motion is introduced to study memory effects in the fluctuations [24]. These memory effects can be embodied by the Hurst exponent *H*, which can be used to measure the long-range correlation and the self-similarity of a time series. The long-range correlation is an important feature of a time-series signal. It reflects the statistical correlation of two data points in a certain time interval and the inherent fluctuation nature of a signal. The self-similarity shows that a time series can be measured on different scales. It can reflect the similarity degree of its fluctuation. Long-range correlation and self-similarity are important properties of nonlinear systems, which are of significance for system modeling and simulation, and system behavior prediction. The Hurst index analysis methods mainly include: power spectrum analysis, rescaled range analysis, and detrended fluctuation analysis.

DFA is a statistical tool that uses *H* to evaluate the long-range correlation of a time series. By calculating the short-long-range correlation characteristics of time-series signals, the trend of a time series can be characterized by fractal properties. DFA is suitable for analyzing non-linear and non-stationary signals, and the fractal structure of a time series can be reduced. By removing the trend components of different orders from a time series, the intrinsic statistical characteristics of the time-series signal are presented accurately.

For a time-series signal *x_m_* (*m* = 1, 2, 3, *L*), the steps of DFA are provided as follows:

(Step 1) Calculate the value $\bar{x}$ by averaging the original series *x_m_*

(1) $$\bar{x}=\frac{1}{L}\sum_{m=1}^{L}x_{m}$$

A new integrated series *y*(*n*) can be obtained as

(2) $$y(n)=\sum_{m=1}^{n}\left(x_{m}-\bar{x}\right),\;n=1,2,3,\ldots ,L$$

(Step 2) Divide *y*(*n*) into equal-length intervals containing *s* points, and the number of the sub-interval can be expressed as

(3) $$L_{s}=\left[L/s\right]$$

where [L/s] represents the integer-valued operation.

(Step 3) Use the least-squares method to fit the data of each sub-interval, and the fluctuation trend *y_f_*(*n*) of sub-interval can be obtained as

(4) $$y_{f}(n)=\sum_{r=0}^{R}a_{r}n^{r}$$

where *a_r_* denotes the fitting *R*-order polynomial coefficient.

(Step 4) Eliminate the fluctuation trend in sub-interval

(5) $$\Delta y_{f}(n)=y(n)-y_{f}(n)$$

(Step 5) Calculate the mean square fluctuation $F_{\tau }{}^{2}(s)$ inside sub-interval *τ*

(6) $$F_{\tau }{}^{2}(s)=\frac{1}{s}\sum_{n=1}^{s}{\left[\Delta y_{f}(n)\right]}^{2}$$

(Step 6) The 2nd-order wave function of the full sequence data can be calculated as

(7) $$F_{q}(s)=\sqrt{\frac{1}{L_{s}}\sum_{\tau =1}^{L_{s}}F_{\tau }{}^{2}(s)}$$

(Step 7) Change the sub-interval length *s* in step 2, and repeat steps 2 to 5 to obtain the full-sequence fluctuation *F_q_*(*s*) as a function of *s*. Fit the fluctuation function by using the least-squares method to obtain a linear function

(8) $$\log_{10}F_{q}(s)=H_{\alpha }\log_{10}s+\log_{10}A$$

where *H_α_* is the Hurst exponent, *A* is a constant calculated by DFA.

The detrended fluctuations function, as shown in Equation (8), has a linear relationship, in which the slope is *H_α_* and the intercept is *log*_10_*A*. Commonly, *H_α_* and *A* are selected as the first and the second principal components, respectively, and these two components are then used as the feature vector to perform feature extraction on the vibration signal. As can be seen, the least-squares method is employed for DFA to fit the detrended fluctuations function. Therefore, a well-fitted result can be obtained when the function is a linear relationship. The DFA method can only extract two features for one vibration signal sequence, and the insufficient number of features would reduce the accuracy of the classification results.

### 3.2. Improved DFA

As shown in step 7, the first-order function can be obtained by using the least-squares method to fit the series of the detrended fluctuations function. Therefore, a well-fitted performance can be achieved when the fluctuation function satisfies the first-order function approximately. However, with the complexity of the fluctuations function increased, the fitting effect of DFA may be decreased. Moreover, in the application of the multiple fault type diagnosis, the two features obtained by DFA are usually insufficient to distinguish all the categories. Therefore, the polynomial curve fitting method is used to deal with the fluctuations function for the improvement of the fitting effect and obtain more fault characteristics. The fitting polynomial can be expressed as

(9) $$Y(X,W)=w_{0}+w_{1}X+w_{2}X^{2}+\ldots +w_{N}X^{N}=\sum_{j=0}^{N}w_{j}X^{j}$$

where X= *log*_10_*s*, Y(X,*W*)= *log*_10_*F_q_*(*s*), *N* is the order of the polynomial, *w*_j_ represents the coefficient of the polynomial. As can be seen, the polynomial function is a nonlinear function of X, while it is a linear function of the polynomial coefficient. The mean square error is usually used as the error function to evaluate the polynomial fitting effect of Equation (9)

(10) $$E(W)=\frac{1}{2}\sum_{v=1}^{V}{\left(Y\left(X_{v},W\right)-Y_{v}\right)}^{2}$$

where *E*(*W*) represents the mean square error, *V* represents the total quantity of the fitted data, *Y_v_* is the actual data value corresponding to X*_v_*.

The purpose of fitting data is to minimize the error function. The polynomial fitting effect for the data would be poor when order *N* is low, that is, underfitting, which cannot represent the objective function well. When the value of *N* is large, the fitting curve would be oscillatory and sensitive to noise data, that is, overfitting. Both underfitting and overfitting cannot represent the objective function well. For the model with a defined complexity, the overfitting problem would be reduced with an increase in data. The regularization method can be used to reduce the influence of overfitting when the model complexity is given and the data scale is fixed. In order to reduce the influence of overfitting, the mean square error function of regularization method can be expressed as

(11) $$\tilde{E}(\varepsilon ,W)=\frac{1}{2}\sum_{v=1}^{V}{\left(Y\left(X_{v},W\right)-Y_{v}\right)}^{2}+\frac{\lambda }{2}||W{||}^{2}$$

where $||W{||}^{2}=W^{T}W=w_{0}^{2}+w_{1}^{2}+\ldots +w_{N}^{2}$, *λ* represents the penalty term which is used to constrain the polynomial coefficient.

Choosing the appropriate value of *λ* according to the complexity of the model has an important impact on the fitting results. *λ* is usually set artificially according to the complexity of the model when the polynomial order *N* is given. Therefore, it is difficult to obtain the optimal coefficient of polynomial fitting by the empirical method. In this paper, the PSO algorithm is employed to optimize the penalty term. PSO was first proposed by Dr. Eberhart and Dr. Kennedy in 1995. This optimization algorithm originated from the research on the predatory behavior of birds. The principle of PSO is to make use of the information shared by the individuals in the group so that the movement of the whole group will evolve from disorder to order in the solving space, so as to obtain the optimal solution of the problem. For the PSO algorithm, each element in the particle swarm represents the possible solution. Through the simple behavior of individual particles and the information interaction within the group, the intelligence of problem-solving is realized. Because of the advantage of simple operation and fast convergence speed, PSO has been widely used in many fields such as function optimization, image processing, and other fields.

**Figure 2:**
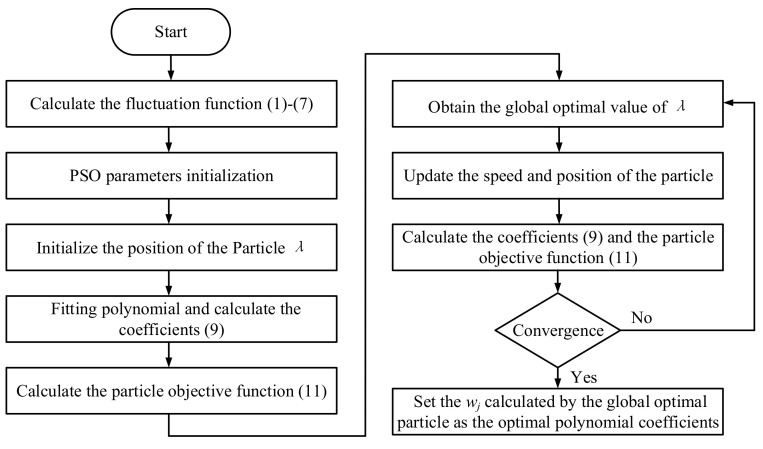
The general flow diagram of the proposed IDFA.

Figure 2 shows the flow diagram of the proposed IDFA method. The fluctuation function is first calculated by Equations (1)–(7). Next, the PSO parameters and the value of the particle *λ* are initialized. The coefficients of the polynomial can be calculated, and the regularized mean square error function is then taken as the particle objective function. The global optimal value of the particle *λ* can be obtained by using the PSO optimization algorithm. Finally, the coefficients calculated by using the optimal value of *λ* can be taken as the optimal polynomial fitting coefficients. The optimal coefficients can be set as the features for the diagnosis of the bearing fault type.

## 4. Multi-Sensor Data Fusion-Based LDA Classifier Design

Vibration sensors mounted on different locations of a rotating machine system can provide complementary information on the health status of the rolling element bearings [35]. On this basis, the LDA classifier based on the multi-sensor data fusion is presented in this paper. The basic idea of the proposed multi-sensor data fusion-based LDA classifier is that one can use the IDFA-extracted feature vectors to fuse and obtain the full training matrix of the LDA classifier. The multi-sensor data fusion approach and the methodology of the LDA algorithm are introduced next.

### 4.1. Multi-Sensor Data Fusion

**Figure 3:**
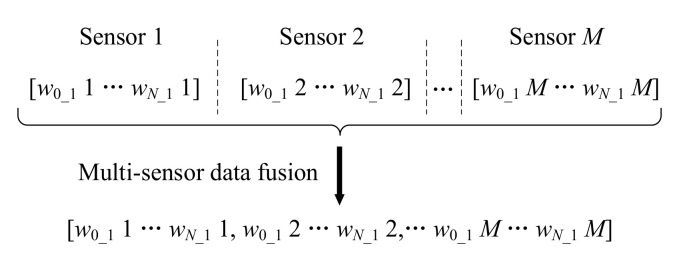
The reconstruction process of the feature vector.

By using IDFA for feature extraction, the vibration signal obtained by one sensor has *N*+1 features, and the two extracted features can be chosen as one feature vector $\left[w_{0\_1}1,w_{1\_1}1,\ldots ,w_{N\_1}1\right]$. Similarly, the feature vector obtained by the *M*-th sensor can be expressed as $\left[w_{0\_1}M,w_{1\_1}M,\ldots ,w_{N\_1}M\right]$. In order to obtain comprehensive information on the health status of bearings, these feature vectors obtained from sensors mounted on different locations should be reconstructed into one feature vector. The reconstruction process of the feature vector is shown in Figure 3.

As shown in Figure 3, the *M* feature vectors can be reconstructed as one feature vector. The complementary information provided by the *M* sensors can be expressed comprehensively by the reconstructed feature vector.

**Figure 4:**
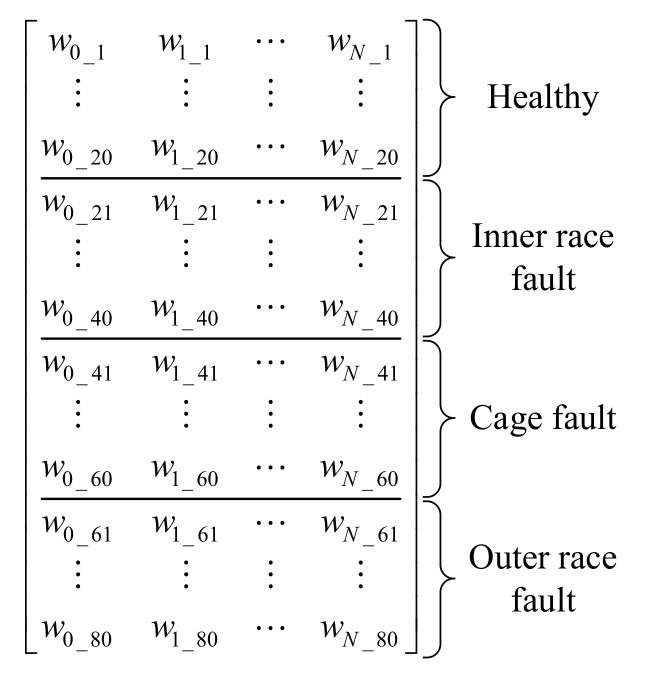
Full training matrix of single-sensor data.

Four classes are studied in this work (healthy bearing and the three fault types), and 20 sets of samples in each class are collected as the training data, so a total of 80 sets of samples are used for training. The full training matrix of a single sensor is shown in Figure 4.

**Figure 5:**
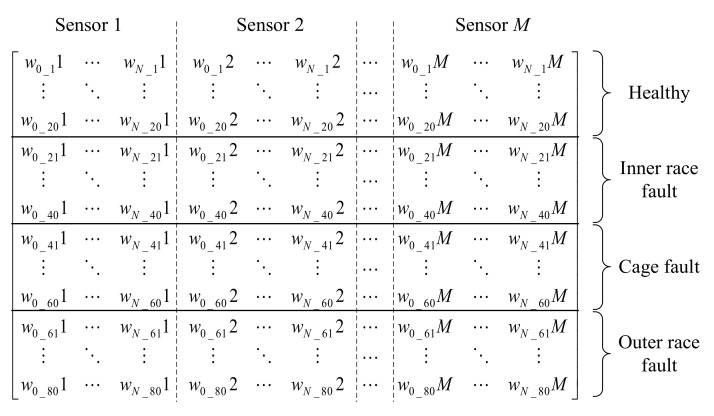
Full training matrix of multi-sensor fusion data.

For the single-sensor classification method, the complementary information provided by other sensors is not contained, so the classification accuracy of the LDA classifier would be limited. Based on Figure 3 and Figure 4, the full training matrix of the proposed multi-sensor fusion method is shown in Figure 5.

As shown in Figure 5, comprehensive information on the health status of the rolling element bearings is expressed by the full training matrix. Therefore, the accuracy of the classification result would be improved by using the full training matrix of multi-sensor fusion data. Two single-axis accelerometers are employed for data fusion in this work.

### 4.2. LDA Classifier

LDA is used to maximize the ratio of the variance between the same and the different classes, so as to achieve the maximum separation between feature sets in each class. For *k*-classes cases, the average vector can be calculated by Equation (12)

(12) $$\mu =\frac{1}{n}\sum_{\forall x}x=\frac{1}{n}\sum_{i=1}^{k}D\mu_{i}$$

where *i* = 1, 2, 3, …, *k*, *x* represents the original data for classification, *n* represents the total number of samples, *D* represents the data amount of *i*th class samples, and *μ_i_* is the center of *i*th class samples.

The intra-class scattering matrix *S_w_* and inter-class scattering matrix *S_b_* of the original data are expressed as

(13) $$S_{w}=\sum_{i=1}^{k}S_{i}^{2}=\sum_{i=1}^{k}\sum_{x_{i}\in \;\mathrm{Class}\;i\;}\left(x_{i}-\mu_{i}\right){\left(x_{i}-\mu_{i}\right)}^{T}$$

(14) $$S_{b}=\sum_{i=1}^{k}n_{i}\left(\mu_{i}-\mu \right){\left(\mu_{i}-\mu \right)}^{T}$$

where the maximum rank of *S_b_* is *k* − 1.

The scattering matrices of the projection data can be written as

(15) $${\tilde{S}}_{w}=V^{T}S_{w}V$$

(16) $${\tilde{S}}_{b}=V^{T}S_{b}V$$

where *V* is the transformation matrix that projects the original data into the low dimensional space.

LDA optimization projection direction is mainly based on the Fisher criterion function. The purpose of LDA is to find the optimal transformation matrix *V* by maximizing the ratio of distance between classes and distance within classes after projection. Therefore, the objective function *J*(*V*) can be expressed as [36]

(17) $$J(V)=\frac{\det\left({\tilde{S}}_{b}\right)}{\det\left({\tilde{S}}_{w}\right)}=\frac{\det\left(V^{T}S_{b}V\right)}{\det\left(V^{T}S_{w}V\right)}$$

The objective function *J*(*V*) can be solved by the Lagrange multiplier method. The Lagrange function is defined as

(18) $$L\left(V,\vartheta \right)=V^{T}S_{b}V-\vartheta \left(V^{T}S_{w}V-M\right)$$

where ϑ is the Lagrange multiplier, and *V* can be obtained from the partial derivation

(19) $$\frac{\partial L\left(V,\gamma \right)}{\partial V}=S_{b}V-\gamma \left(S_{W}V\right)$$

Let the partial derivative be 0

(20) $$S_{b}v=\gamma S_{w}v,\;\left(\gamma =\frac{v^{T}S_{b}v}{v^{T}S_{w}v}\right)$$

where *γ* is the eigenvalue corresponding to the eigenvector *v*. In the solution of Equation (20), there are at most *k* − 1 linearly independent vectors, which can be expressed as *v*_1_, *v*_2_, …, *v_k*−1*_*.

Therefore, the optimal projection direction of LDA can be obtained by generalized eigenvalue decomposition of Equation (20). Then, the optimal transformation matrix *V* of D dimension subspace can be obtained. The *k*-classes linear discriminant function is given by (21)

(21) $$C_{j}\left(X_{j}\right)=V_{j}^{T}X_{j}+v_{j0}=v_{j_{1}}x_{j_{1}}+v_{j_{2}}x_{j_{2}}+\ldots +v_{j_{D}}x_{j_{D}}+v_{j_{0}}$$

where *j* = 1, 2, 3, …, *k* − 1, $X_{j}=[x_{j_{1}},x_{j_{2}},\ldots x_{j_{D}}]$ is the *D* dimension vector of the *j* class sample *X_j_*, $V_{j}^{T}=[v_{j_{1}},v_{j_{2}},\ldots v_{j_{D}}]$ is the coefficient matrix of the *j* class, which can be calculated by Equation (17), *v_j0_* represents the threshold value of the *j* class sample classification.

In the training phase, for each training sample *X_j_* belonging to the *j* class, the coefficient matrix is obtained by training to make *C_j_*(*X_j_*) larger than all other classes. In order to classify the unknown samples, the coefficient matrix calculated in the training phase will be used to calculate the discriminant function of the tested sample *X_t_*. If one kind of linear discriminant function of the tested sample is larger than any other linear discriminant function, the test sample can be divided into this kind. That is, if Equation (22) is satisfied, the tested sample belongs to *p* class.

(22) $$C_{p}\left(X_{t}\right)\geq C_{q}\left(X_{t}\right)\;\forall p\neq q$$

## 5. Experimental Results and Discussion

### 5.1. Experimental Setup

**Figure 6:**
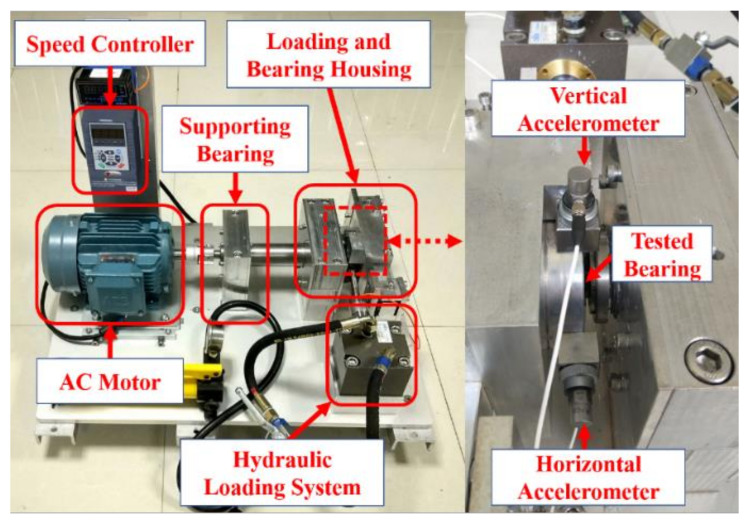
Bearing test rig and the location of sensors adapted from [37,38].

In most of the existing literature, fault vibration signals are commonly collected by seeding bearing faults. However, the real vibration signals of fault bearings are not the same as the data collected by setting the fault. This paper uses the full life-cycle vibration data of Xi’an Jiaotong University to verify the proposed fault diagnosis method [37]. Figure 6 depicts the accelerated life test rig for recording vibration signals [38].

**Figure 7:**
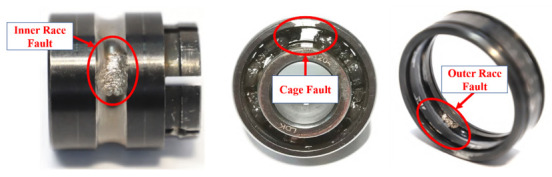
Three bearing faults generated in the accelerated life experiment.

As shown in Figure 6, the test rig consists of an AC motor, speed controller, supporting bearing, hydraulic loading system, and loading and bearing housing. Accelerated life tests of various types of rolling bearings under different working conditions can be carried out by using the test bench, so the life-cycle monitoring data of tested bearings can be collected. The adjustable working conditions of the platform mainly include loading force and rotational speed. The loading force is generated by the hydraulic loading system and can be loaded on the bearing housing of the tested bearing. The rotational speed can be adjusted by the speed controller of the AC motor. The LDK UER204 rolling bearing is tested, and the parameters are shown in Table 1. Figure 7 shows the three bearing faults generated in the accelerated life experiment.

As shown in Figure 7, three damaged bearings with inner race, cage, and outer race faults were examined in this work. The experimental data with different levels of fault severity related to its evolution would be generated during the data collection process. The experimental procedure adopted in this work may make the proposed method appear more efficient in detecting a particular type of defect, because the same bearing fault types (different bearing, different fault shape, etc.) of experimental data were not collected and compared.

For vibration signal acquisition, two PCB 352C33 single-axis acceleration sensors were fixed to the horizontal and vertical directions on the bearing housing, respectively. A data dynamic acquisition device DT9837 was used to collect vibration data. In the accelerated life experiment, the healthy bearing was placed in the bearing housing. During the testing, the hydraulic loading system provided a constant load of 11 kN, and a rotation frequency of 2250 rpm was generated by the AC motor. The sampling frequency was set to 25.6 kHz.

Vibration signals were collected until the maximum amplitude of the fault signal was more than 10 times the healthy value. Three different fault types were generated: inner race fault, cage fault, and outer race fault. The tested bearing information of the accelerated life experiment is provided in Table 2. As shown in Table 2, the total samples contain all the vibration information of the bearing from early to late failures. Therefore, for each type of fault, 40 samples of vibration signals with different fault degrees were employed in this work to validate the effectiveness of the proposed method.

### 5.2. Feature Extraction

**Figure 8:**
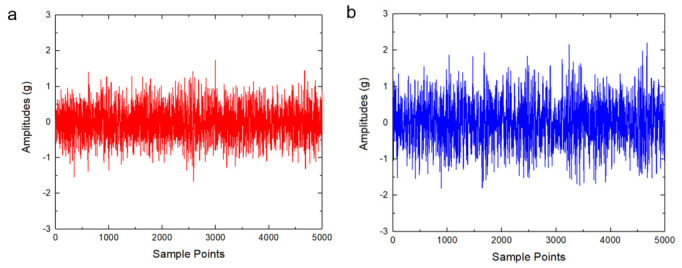
The vibration signals of a healthy bearing. (**a**) The signal of a horizontal accelerometer. (**b**) The signal of a vertical accelerometer.

Figure 8 shows vibration signals obtained from the two accelerometers working under healthy conditions. Each original series contains 5000 points. From Figure 8, one can see that, in the healthy state, the vibration signal is stable. The amplitude of the vibration acceleration in the vertical direction is 1 g, and the amplitude in the horizontal direction is 1.5 g. The difference of the amplitudes in the two directions is caused by the uneven load application. Overall, the vibration trends in vertical and horizontal directions are basically the same.

**Figure 9:**
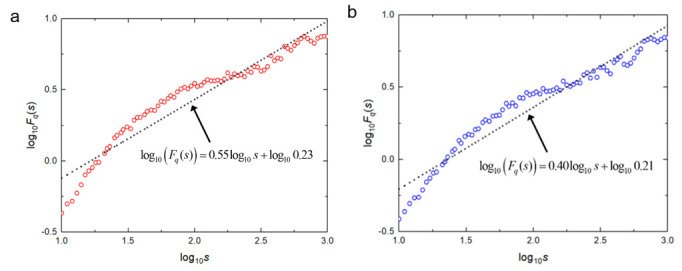
Detrended fluctuation analysis (DFA) results of the healthy vibration signals. (**a**) The DFA result of a horizontal accelerometer. (**b**) The DFA result of a vertical accelerometer.

The corresponding DFA curves of the healthy bearing are shown in Figure 9. According to the calculation approach in [24], the corresponding DFA curves were calculated for a maximum of 81 values of *log*_10_*s*, as the red and the blue circles show in Figure 9a,b, respectively. The corresponding least-squares fitted results of DFA curves are also shown in Figure 9. The fitted curves *log*_10_*Fq*(*s*) representing the various conditions can be used for feature extraction, and then can be employed in conjunction with the LDA classifier aiming at fault classification.

As shown in Figure 9, The fluctuant trend of the two DFA curves is basically the same. The first principal components *H_α_* of the vertical and the horizontal vibration signals are 0.55 and 0.40, respectively. Meanwhile, the second principal components *A* have similar values of 0.23 and 0.21. This shows that the difference between the two features in the horizontal direction is not obvious. Therefore, the signals obtained by the two sensors are not distinguishable on the second principal component, and the difference is mainly reflected by the first. The DFA curve of the healthy bearing is approximately in line with the positive proportional function. Therefore, a well-fitted result can be achieved by using the least-squares method.

**Figure 10:**
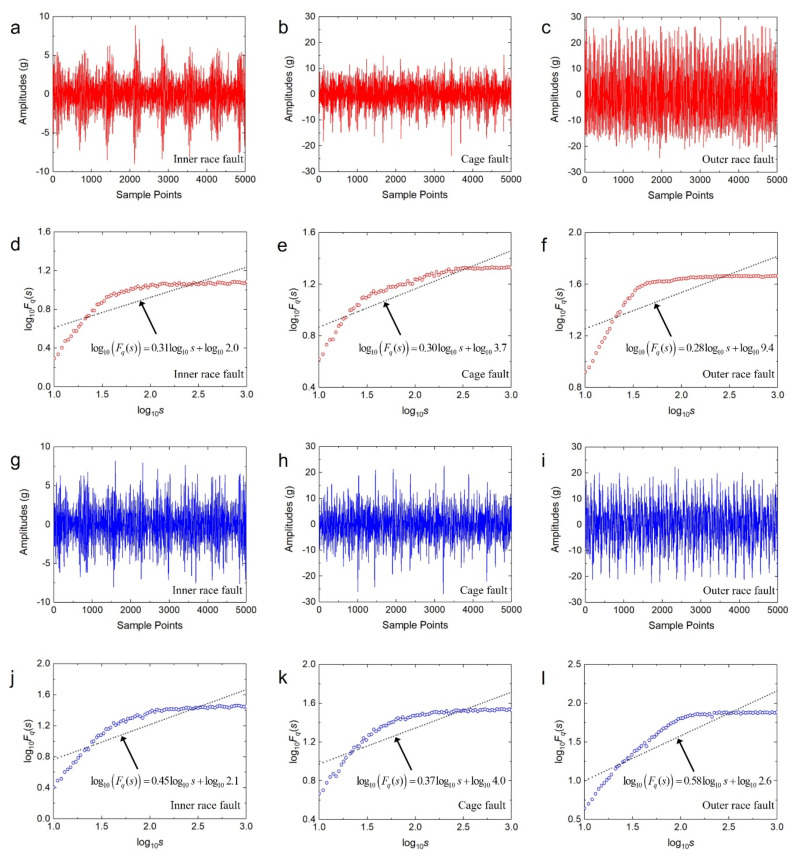
The vibration signals and DFA results of three fault types. (**a**–**f**) Obtained from the horizontal accelerometer. (**g**–**l**) Obtained from the vertical accelerometer.

Figure 10 shows the results of vibration accelerated life experiments and corresponding DFA curves of the two acceleration sensors under three faults conditions. Compared with the healthy vibration acceleration waveform in Figure 8, the vibration signals of the bearings with faults have obvious changes in both amplitude and shape. The vibration signals of bearings with faults contain a large number of periodic pulse signals. These changes can be reflected through DFA analysis. From Figure 10d–f, one can see that the first components are 0.31, 0.30, and 0.28, and the second components are 2.0, 3.7, and 9.4, respectively. The values of the first components are smaller than 0.55 of the healthy bearing, and the values of the second components are larger than 0.23 of the healthy signal. Therefore, both the first and the second components can be used as features to distinguish the healthy bearing and the bearings with faults. For the horizontal sensor, because the three values of the first components are similar, the three faults are not distinguished clearly on the first components, while the second components can be used to distinguish the fault type well. From Figure 10j–l, one can see that the first components are 0.45, 0.37, and 0.58, and the second components are 2.1, 4.0, and 2.6, respectively. Note that there is an overlap interval between the values of the first components and the value 0.40 of the healthy signal, which may cause misdiagnosis. The values of the second components are significantly larger than 0.21 of the healthy signal. The second components can be used as features for determining whether a fault of bearings has occurred. For the vertical sensor, the three types of faults are clearly distinguished on the first components, and the first components can be used to distinguish the fault type well. Compared with the healthy bearing, the fluctuation function of the fault vibration signal is not a positive proportional function. Therefore, the least-squares method is ineffective in fitting the fluctuation function of the faulty bearing.

As can be seen, only two features can be obtained by using DFA. Although the features obtained from the healthy bearing and the fault vibration signal are obviously different, the features extracted from the various fault signals are overlapped, which would cause the weakening of the difference of the fault features. In addition, the least-squares method cannot fit the fluctuation function of the fault vibration signal effectively, which would lead to the extracted fault features that cannot reflect the fluctuation trend of the vibration signals. Therefore, the fault features extracted by DFA have limitations in the diagnosis applications of multiple bearing fault types, such as when the feature difference of various faults is not obvious, and the fitting result of the fluctuation function is poor. These would affect the accuracy of fault feature extraction and lead to the reduction of fault diagnosis accuracy.

**Figure 11:**
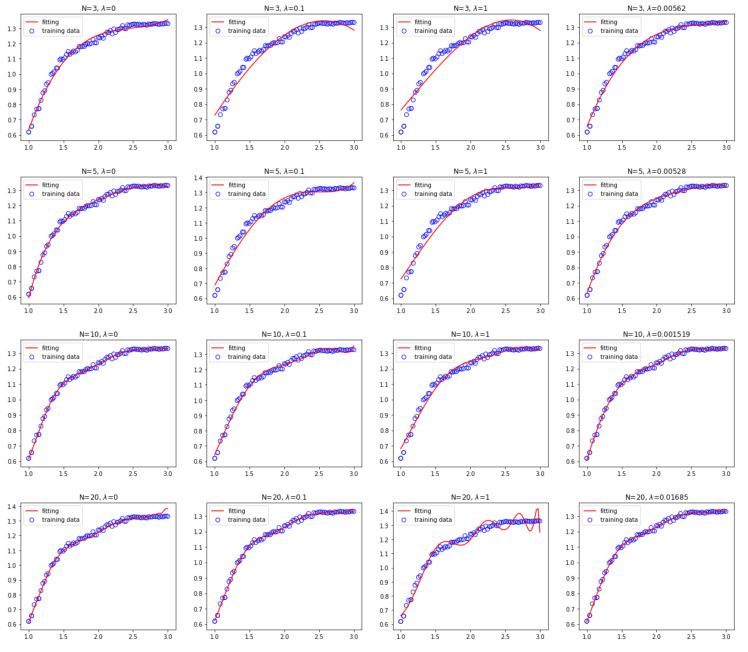
The fitting results of the third-, fifth-, 10th-, and 20th-order fitting polynomials when *λ* is equal to 0,0.1,1 and the PSO optimal value, respectively.

In order to verify the effectiveness of the proposed IDFA feature extraction method, the fitting results of the fluctuation function are presented. Figure 11 shows the fitting results of the third-, fifth-, 10th-, and 20th-order fitting polynomials when *λ* is equal to 0, 0.1, 1 and the PSO optimal value, respectively.

From Figure 11, one can see that, for the third-order fitting, the fitting curve with *λ* = 0 has better fitting performance in the front part, while the fitting effect in the latter part becomes worse. The fitting results of *λ* = 0.1 or *λ* = 1 are significantly worse. When *λ* equals the optimal value of PSO, the best fitting effect can be achieved. For the fifth- and tenth-order fitting, the fitting effect is basically the same when *λ* = 0 or when λ equals the optimal value, which can realize the fitting of the fluctuation function well, and the fitting effect is better than that of *λ* = 0.1 or *λ* = 1. For the 20th-order fitting, the best fitting performance can be obtained when *λ* = 0.1 or when λ set as the optimal value. The fitting curve with *λ* = 0 has better fitting effect in the front part, however, the fitting effect in the latter part becomes worse. The fitting curve fluctuates obviously when *λ* = 1, which is mainly caused by the excessive value of λ. Therefore, the fitting performance when λ equals the optimal value is better than that when λ is set as a fixed value. Therefore, the fitting effect can be improved by using the IDFA proposed in this paper for feature extraction.

### 5.3. LDA Training

**Figure 12:**
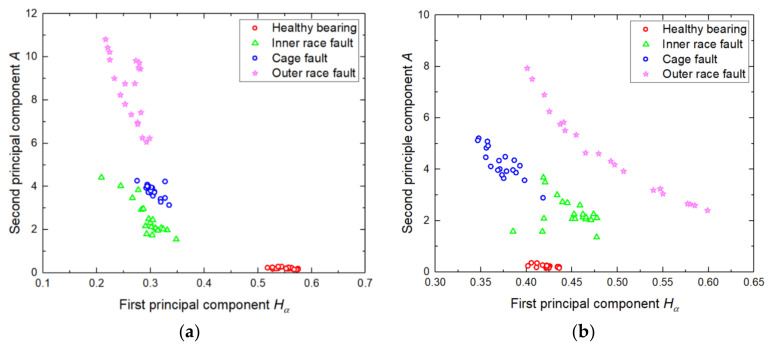
Feature vectors obtained by DFA. (**a**) Obtained from the horizontal sensor. (**b**) Obtained from the vertical sensor.

For each type of fault, 40 sequences of vibration signals were collected. Therefore, a total of 160 samples with different fault types were studied in this paper. The 160 samples were split into two groups: 50% for training and 50% for testing. The training sets were obtained from the DFA-extracted and IDFA-extracted feature samples, respectively. The 80 DFA-extracted feature samples used for training are shown in Figure 12.

As shown in Figure 12, the DFA results of 20 samples of each fault type fluctuate within a certain range. Among the four classes, the distinguishing characteristics between the healthy signal and the outer ring fault signal are obvious, while the characteristics of the inner ring fault and the cage fault signal have a cross section. In Figure 12a, the first and second principal components of the healthy signal are clearly different from the other three types of faults. The faults occurred in the inner ring, the cage, and the outer ring are distributed in the range of [0.2, 0.4] on the first component. This means that the first component cannot distinguish the three faults well. The characteristics of the three faults are not obvious on the second component. In Figure 12b, the characteristics of outer ring faults are more widely distributed, and this characteristic of the distribution is clearly distinguished from the healthy bearing, inner ring fault, and cage fault. One can see that the second principal component of the healthy signal remains low. The inner race fault and the cage fault have a cross range around the value 0.4 of the first component and the value 3 of the second component, which may cause misclassification.

**Figure 13:**
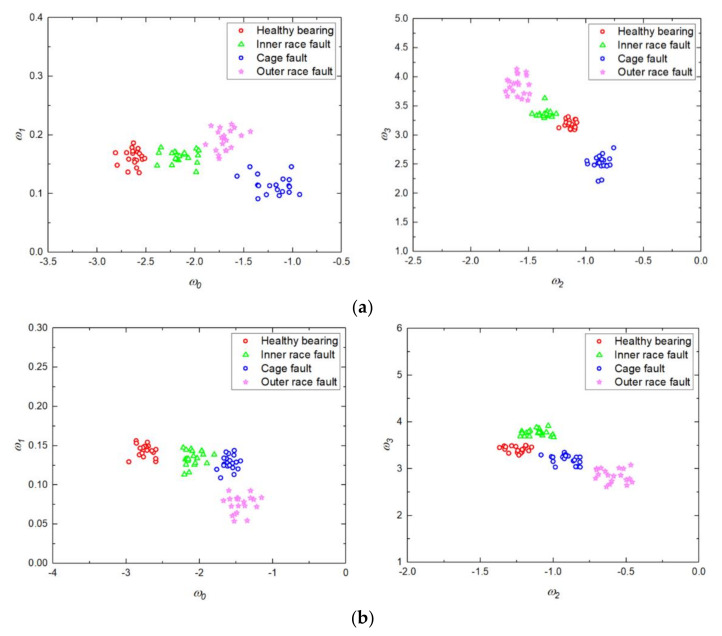
Feature vectors of a bearing obtained by IDFA. (**a**) Obtained from the horizontal sensor. (**b**) Obtained from the vertical sensor.

As for the reason that DFA employs the least-squares method to fit the fluctuation function, the fitting effect of DFA becomes worse when the fluctuation function is complex, so the accuracy of fault feature extraction would be affected. Moreover, the DFA method can only obtain two features (*H_α_* and *A*), and the insufficient number of features usually results in a poor classification accuracy in the diagnosis application of multiple fault types. In this paper, IDFA is used to extract the fault features of time-domain vibration signals, the third-order polynomial is used to fit the fluctuation function, and the coefficients of the third-order fitting polynomial are calculated by using the *λ* value optimized by PSO. Therefore, four features (*w*_0_, *w*_1_, *w*_2_, and *w*_3_) can be obtained from the vibration sequence. The 80 IDFA-extracted feature samples used for training are shown in Figure 13.

As shown in Figure 13a, the values of feature *w*_1_ of the four categories are small, and feature *w*_1_ is concentrated around 0.15. Feature *w*_1_ of the healthy bearing and the inner race fault have a high coincidence. Therefore, the four types cannot be discriminated against by feature *w*_1_ effectively. In Figure 13b, the values of feature *w*_1_ of the four categories are close to 0. The regions of feature *w*_1_ of inner race fault and cage fault are highly coincident. Thus, feature *w*_1_ has little differentiation from the four types, while *w*_0_, *w*_2_, and *w*_3_ can distinguish the four categories effectively. Because of the values of extracted feature *w*_1_ are small, and the discrimination of feature *w*_1_ for the four types is limited, and the differences of IDFA-extracted features are mainly reflected in *w*_0_, *w*_2_, and *w*_3_.

**Figure 14:**
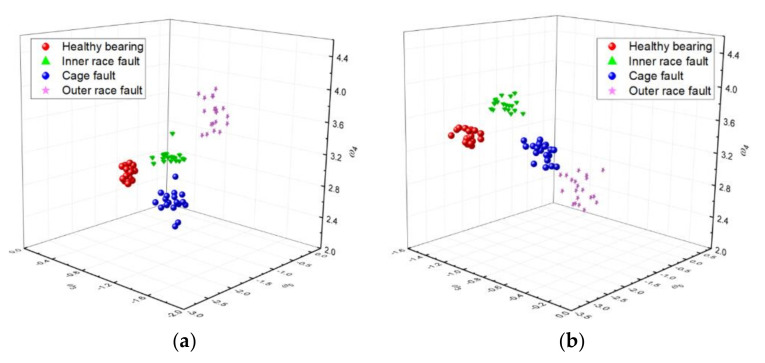
Feature vectors in 3D space obtained by IDFA. (**a**) Obtained from the horizontal sensor. (**b**) Obtained from the vertical sensor.

Figure 14 shows the distribution of the extracted features *w*_0_, *w*_2_, and *w*_3_ in three-dimensional space. As can be seen from Figure 14, the coincident region of the inner race and the cage fault features has been obviously reduced compared with Figure 12. Therefore, the distinguishing degree of the four classes of fault features is improved by using IDFA for feature extraction.

The 80 samples shown in Figure 12 and Figure 13 were used for the generation of the training matrix (as shown in Figure 4 and Figure 5) to train the LDA algorithm. Then, the rest of the 80 testing samples can be classified by the trained LDA classifier.

### 5.4. Classification Accuracy

Next, the classification accuracy of the trained LDA classifier under different training matrix will be discussed to validate the effectiveness of the proposed multi-sensor fusion-based fault diagnosis method.

Table 3 shows the classification results using the horizontal sensor (HS) and the vertical sensor (VS), respectively. As can be seen from Table 3, by using DFA for feature extraction, the classification accuracies of HS for the healthy bearing and the outer race fault are 100% and 95%, respectively. The classification accuracies of VS for the healthy bearing and the outer race fault are 95% and 100%, respectively. Meanwhile, the accuracy of the DFA method on both inner ring and cage faults is lower than 90%. When employing the IDFA method, the classification accuracies of the healthy bearing and the outer race fault are both higher than the other two classes. Therefore, the single-sensor method has a high accuracy rate for the classification of the healthy bearing and the bearing with an outer ring fault, while the accuracy on both inner ring and cage faults is unsatisfied. Moreover, when IDFA is used for feature extraction, the classification accuracies of HS and VS for the three categories (healthy, inner race fault, and cage fault) are higher compared with the DFA method. Overall, an accuracy of 90% (or 91.3%) can be achieved by the DFA method, and the classification accuracy of HS and VS can reach 95.0% and 97.5%, respectively, by using the IDFA-based feature extraction method.

Table 4 shows the fault classification results using the sensor fusion diagnosis method proposed in this paper. From Table 4, by using DFA for feature extraction, one can see that the classification accuracy of the healthy bearing and the bearings with inner ring faults and outer ring faults can reach 100%, and the classification accuracy for cage faults can achieve 95%. The classification accuracy of the IDFA-based feature extraction method can reach 100% for the four classes. Overall, the sensor fusion classification accuracy of DFA and IDFA is 98.8% and 100%, respectively. The classification accuracy of the four categories when using the proposed diagnosis approach all reach 100%. However, the proposed method may obtain a lower classification accuracy while detecting the same type of defect on different bearings, because limited types of data have been considered in the experiment.

Table 5 shows the comparison of the proposed multi-sensor fusion diagnosis method with the classification results of the single-sensor method. It can be seen from Table 5 that, by using the DFA-based feature extraction method, the classification accuracy of the sensor-fusion diagnosis method is 8.8% and 7.5% higher than that of HS and VS, respectively. By using the IDFA-based feature extraction method, the classification accuracy of the sensor fusion diagnosis method is 5% and 2.5% higher than that of HS and VS, respectively. Moreover, the classification accuracy of the proposed IDFA and multi-sensor-fusion diagnosis method can reach 100%.

## 6. Conclusions

This paper has presented a fault diagnosis method for rolling element bearings based on IDFA and multi-sensor data fusion. By using the proposed IDFA-based feature extraction method, the fault features of bearings are extracted effectively. First, the corresponding fluctuation function calculated from the time-domain vibration signals is obtained. Next, PSO is employed for the parameter optimization to obtain the optimal fitting polynomial of the fluctuation function. The polynomial coefficients are then selected as the fault features which can be classified by the classifier. A multi-sensor data fusion classifier based on LDA is also presented for the classification. In particular, the data obtained from two single-axis accelerometers were analyzed to improve classification accuracy. The extracted features are then reconstructed into one feature vector to obtain the full training matrix of the LDA classifier. Three faults were discussed: inner race, cage, and outer race fault. The effectiveness of the proposed diagnosis method was validated using the accelerated life experimental data. The validation results have shown that an accuracy of 90% (or 91.3%) for rolling element bearings was achieved by the DFA and single-sensor approach. The classification accuracy of the IDFA and single-sensor method can reach 95% (or 97.5%). Furthermore, 100% diagnostic accuracy can be achieved by applying the proposed IDFA and multi-sensor data fusion method. An initial study which considered a limited fault category has been conducted in the experimental process. The limitation of this work is that limited types of data have been considered, so that the classification accuracy of the proposed method may be influenced when using the data of different bearings. Future work will be focused on the detection of other bearings and fault diagnosis under non-stationary conditions.

## Figures and Tables

**Table 1:** Dimensional parameters of LDK UER204.

Parameter Name	Value
Inside diameter	29.30 (mm)
Outside diameter	39.80 (mm)
Pith diameter	34.55 (mm)
Ball diameter	7.92 (mm)
Number of balls	8

**Table 2:** Tested bearing information of the accelerated life experiment.

Type of Fault	Total Samples	Actual Life
Inner race	491	8.18 (h)
Cage	533	8.83 (h)
Outer race	339	5.65 (h)

**Table 3:** Single-sensor classification results.

Type of Fault	Feature Extraction Method	Tested Samples		Correctly Classified Samples		Classification Accuracy (%)	
		HS	VS	HS	VS	HS	VS
Healthy	DFA	20	20	20	19	100.0	95.0
	IDFA	20	20	20	20	100.0	100.0
Inner race	DFA	20	20	17	17	85.0	85.0
	IDFA	20	20	18	19	90.0	95.0
Cage	DFA	20	20	16	17	80.0	85.0
	IDFA	20	20	19	19	95.0	95.0
Outer race	DFA	20	20	19	20	95.0	100.0
	IDFA	20	20	19	20	95.0	100.0
Overall	DFA	80	80	72	73	90.0	91.3
	IDFA	80	80	76	78	95.0	97.5

**Table 4:** Multi-sensor classification results.

Type of Fault	Feature Extraction Method	Tested Samples	Correctly Classified Samples	Classification Accuracy (%)
Healthy	DFA	20	20	100.0
	IDFA	20	20	100.0
Inner race	DFA	20	20	100.0
	IDFA	20	20	100.0
Cage	DFA	20	19	95.0
	IDFA	20	20	100.0
Outer race	DFA	20	20	100.0
	IDFA	20	20	100.0
Overall	DFA	80	79	98.8
	IDFA	80	80	100.0

**Table 5:** The compared results of single-sensor and multi-sensor classification.

Sensor	Feature Extraction Method	Tested Samples	Correctly Classified Samples	Classification Accuracy (%)
HS	DFA	80	72	90.0
	IDFA	80	76	95.0
VS	DFA	80	73	91.3
	IDFA	80	78	97.5
Multi-sensor	DFA	80	79	98.8
	IDFA	80	80	100.0
